# CCAAT/Enhancer Binding Protein β Is Dispensable for Development of Lung Adenocarcinoma

**DOI:** 10.1371/journal.pone.0120647

**Published:** 2015-03-13

**Authors:** Yi Cai, Ayako Hirata, Sohei Nakayama, Paul A. VanderLaan, Elena Levantini, Mihoko Yamamoto, Hideyo Hirai, Kwok-Kin Wong, Daniel B. Costa, Hideo Watanabe, Susumu S. Kobayashi

**Affiliations:** 1 Department of Medicine, Beth Israel Deaconess Medical Center, Harvard Medical School, Boston, Massachusetts, United States of America; 2 Department of Pathology, Beth Israel Deaconess Medical Center, Harvard Medical School, Boston, Massachusetts, United States of America; 3 Institute of Biomedical Technologies, National Research Council (CNR), Pisa, Italy; 4 Department of Transfusion Medicine & Cell Therapy, Kyoto University Hospital, Kyoto, Japan; 5 Department of Medical Oncology, Dana-Farber Cancer Institute, Boston, Massachusetts, United States of America; 6 Cancer Program, Broad Institute of MIT and Harvard, Cambridge, Massachusetts, United States of America; 7 Harvard Stem Cell Institute, Cambridge, Massachusetts, United States of America; Cincinnati Children's Hospital Medical Center, UNITED STATES

## Abstract

Lung cancer is the leading cause of cancer death worldwide. Although disruption of normal proliferation and differentiation is a vital component of tumorigenesis, the mechanisms of this process in lung cancer are still unclear. A transcription factor, C/EBPβ is a critical regulator of proliferation and/or differentiation in multiple tissues. In lung, C/EBPβ is expressed in alveolar pneumocytes and bronchial epithelial cells; however, its roles on normal lung homeostasis and lung cancer development have not been well described. Here we investigated whether C/EBPβ is required for normal lung development and whether its aberrant expression and/or activity contribute to lung tumorigenesis. We showed that C/EBPβ was expressed in both human normal pneumocytes and lung adenocarcinoma cell lines. We found that overall lung architecture was maintained in *Cebpb* knockout mice. Neither overexpression of nuclear C/EBPβ nor suppression of *CEBPB* expression had significant effects on cell proliferation. C/EBPβ expression and activity remained unchanged upon EGF stimulation. Furthermore, deletion of *Cebpb* had no impact on lung tumor burden in a lung specific, conditional mutant EGFR lung cancer mouse model. Analyses of data from The Cancer Genome Atlas (TCGA) revealed that expression, promoter methylation, or copy number of *CEBPB* was not significantly altered in human lung adenocarcinoma. Taken together, our data suggest that C/EBPβ is dispensable for development of lung adenocarcinoma.

## Introduction

Lung cancer is one of the major cancers with more than 220,000 new cases annually in the United States [1]. Lung cancer is histologically classified into two categories: small-cell lung cancer (SCLC) and non-small-cell lung cancer (NSCLC). NSCLC is further classified into three major categories: squamous-cell carcinoma (approximately 30% of all lung cancers), large-cell carcinoma (10–15%) and adenocarcinoma (30–40%) [2]. Although tobacco smoking is considered the dominant cause of lung cancer, in particular SCLC and squamous-cell carcinoma, approximately 10% of lung cancers occur in never smokers [3]. At a molecular level, somatic mutations that inactivate tumor suppressors (e.g., p53) or constitutively activate signaling molecules (e.g., Kras and epidermal growth factor receptor [EGFR]) have been identified during the last three decades. Additional factors include epigenetic changes and altered expression of microRNA [4, 5]. These oncogenic events lead to changes in transcription patterns, resulting in a block in differentiation, suppression of apoptosis, and uncontrolled proliferation [6].

Given that normal lung development is tightly and precisely controlled by complex transcriptional networks, it has been postulated that deregulation of crucial transcription factors can lead to tumorigenesis [7]. Recently, we and others have shown that C/EBPα, a transcription factor essential for proper lung development, is required for differentiation from alveolar type II (AT-II) to alveolar type I (AT-I) cells and that lack of C/EBPα promotes lung adenocarcinoma development [8, 9]. As re-expression of C/EBPα leads to proliferation arrest, differentiation, and increased apoptosis accompanied by morphological changes, it suggests that loss of C/EBPα may contribute to lung cancer development in humans [10, 11]. However, roles of other members of C/EBP family on lung development and tumorigenesis have not been well characterized.

C/EBPβ is a critical regulator of proliferation and/or differentiation in multiple cells/tissues, including myeloid cells, liver, adipose tissues, the immune system, mammary gland and uterus [12–18]. C/EBPβ is known to have three isoforms, which are products of a single mRNA by a leaky ribosomal scanning mechanism [19]. In humans, the full protein and liver-enriched activator protein (LAP) act as activators, whereas liver-enriched inhibitory protein (LIP), the N-terminal truncated form LIP can attenuate the transcriptional activity as it lacks part of the transactivation domain [20]. Therefore, the ratio of their isoforms can determine transcriptional activity [20].

In rodents, C/EBPβ is expressed in alveolar epithelial cell [21] and the bronchiolar epithelium [22]. It has been shown that mice lacking C/EBPβ show no abnormalities in lung function or morphology at perinatal stage [23, 24]; however, its role on adult lung homeostasis or lung cancer development has not been well characterized. Here we sought to determine whether C/EBPβ plays an important role on lung maintenance at adult stage and whether aberrant expression/activity of C/EBPβ contributes to lung cancer development, specifically in a lung adenocarcinoma model.

## Materials and Methods

### Cell lines and culture

All cell lines used in this study (293T, A427, A549, NCI-H125, NCI-H358, NCI-H460, NCI-H1299, NCI-H1395, NCI-H1650, NCI-H1755, NCI-H1975, NCI-H3255, HCC827, PC9, and BEAS-2B cells) were purchased from the American Type Culture Collection. To examine the expression of C/EBPβ upon EGF exposure, A549 and NCI-H1975 were plated at 1x10^5^ cells per mL in 6-well plates and allowed to grow overnight, and then cells were treated with 100 ng/mL EGF in the presence or absence of either 1 μM erlotinib or 1 μM afatinib for 24 hours. For the generation of the cell lines with conditional C/EBPβ expression, NCI-H358 cells were transfected with pBabePuro vector or pBabePuro C/EBPβ-ER and puromycin resistant clones were selected as previously described [25]. Cells were maintained in phenol red free RPMI1650 supplemented with 10% charcoal-dextran–stripped fetal bovine serum. The presence of the fusion protein C/EBPβ-ER was screened by western blot analysis using C/EBPβ antibody (C-19: sc-150). To generate cells transduced with shRNA-mediated suppression of *CEBPB*, NCI-H358 and NCI-H1975 cells were retrovirally infected with shRNA constructs against *CEBPB* (pNa-ntxC/EBPβ) or luciferase gene (control) and sorted for GFP positive cells as previously described [26].

### Mice

The protocol was approved by the Institutional Animal Care and Use Committee at Beth Israel Deaconess Medical Center (Protocol Number: 022–2012). EGFR-L858R-T790M (EGFR^TL^)/CCSP-rtTA bi-transgenic mice and C/EBPβ knockout mice were previously reported [27, 28]. EGFR^TL^ and CCSP-rtTA genotyping were performed as described previously [27]. C/EBPβ knockout mice were genotyped by using following primers: Forward primer; 5’ GGC AGC TGC TTG AAC AAG TTC 3’, Reverse primer 1; 5’ GGC AGC TGC TTG AAC AAG TTC 3’, Reverse primer 2; 5’ CAT CAG AGC AGC CGA TTG TC 3’. PCR reactions were done as follows: 94°C denaturation for 5 min, followed by 35 cycles of 94°C for 40 sec, 58°C for 40 sec, 72°C for 40 sec, followed by a 10 min extension at 72°C. Wildtype and knockout alleles generate 221 bp and 396 bp, respectively. To induce EGFR^TL^ expression, mice were fed with a doxycycline diet (Harlan Laboratories) up to 12 weeks. Lungs were isolated from treated mice which were euthanized by CO_2_. Maximum tumor volume did not exceed 1,000 mm^3^. A total of 39 mice were used in this study.

### Cell lysate preparation and Western blotting

Cell lysates were prepared as previously described [29, 30]. Briefly, cells were washed with PBS and centrifuged at 1500 rpm for 5 minutes. Cell pellets were resuspended in 30–60 μL cell lysis buffer (20 mM Tris-HCl (pH 7.5), 150 mM NaCl, 1 mM EDTA, 1 mM EGTA, 1% Triton, 1 mM β-glycerophosphate, 1 mM Na_3_VO_4_, 1 mM NaF, Protease inhibitor cocktail set III (EMD Millipore), and 1 mM phenylmethanesulfonyl fluoride). Lysates were cleared by centrifugation (14,000 rpm for 15 minutes in a pre-cooled centrifuge) and boiled with SDS sample buffer for 3 minutes. Protein lysates (20–40 μg) were subjected to SDS polyacrylamide gel electrophoresis and blotted on to PVDF membranes (Millipore). We purchased C/EBPβ antibody (C-19) from Santa Cruz Biotechnology (sc-150) and β-actin antibody from Sigma-Aldrich (A3854). Quantitative analysis of relative protein was performed using ImageQuant software (GE Healthcare).

### Immunohistochemical analysis

Lungs isolated from mice were fixed in 10% neutral buffered formalin, embedded in paraffin, sectioned, and stained with H&E. Immunohistochemical staining was performed on formalin-fixed paraffin sections after antigen retrieval, with the antibodies against TTF-1 (Abcam: ab40880) followed by incubations with biotinylated secondary antibody (Jackson ImmunoResearch), advin-biotin complex (Vector Laboratories), and 3, 3’-diaminobenzidine (Sigma-Aldrich).

### Immunofluorescence analysis

NCI-H358 cells expressing pBabePuro vector or pBabePuroC/EBPβ-ER were treated with 1 μM β-estradiol or 0.1% ethanol (vehicle) for 24 hours and stained with anti ERα antibody (Santa Cruz Biotechnology: sc-542) and visualized by Alexa Fluor 488 (Life Technologies). The slides were mounted in Vectashield mounting medium containing DAPI (Vector Laboratories).

### Luciferase reporter gene assay

To measure C/EBPβ transactivation, A549 cells and NCI-H1975 cells were plated at 1x10^5^/mL in 24-well plate and incubated overnight. The following day, cells were transiently transfected with 500 ng *C/ebpb0*.*3-*luc reporter (a gift from Dr. Daniel Tenen) [25] and 10 ng pCMV-*Renilla* plasmid using 1.0 μL TransIT-X2 reagent (Mirus Bio LLC) according to the instructions. The medium was replaced by fresh medium containing 100 ng/mL EGF (Peprotech) or PBS after 6 hours of incubation. The cells harvested with 100μL Passive Lysis Buffer Reporter after 24 hours of treatment with EGF (or PBS) were subjected to reporter assays using the Dual-Luciferase Assay reporter system (Promega. Luciferase activities were normalized against *Renilla* luciferase activity, and the relative luciferase activity was calculated against PBS control.

### Cell Growth Assay

Cell growth was assessed by CellTiter 96 AQueous One solution proliferation kit (Promega) according to manufacturer’s instruction. Briefly, cells were plated in 96 well plates at 2,000 cells/100 μl/well and incubated in 5% CO_2_ for 72 hours. Twenty μl/well of CellTiter 96 AQueous One solution was added and incubated in 5% CO_2_ for 1–4 hours. The absorbance at 490 nm was measured with the Victor 3 microplate reader (PerkinElmer).

### Expression data analyses

Expression datasets from normal human tissues were obtained through Genotype-Tissue Expression (GTEx) project (https://www.gtexportal.org/home) and the distribution of *CEBPB* expression for each tissue type was depicted with a box plot and rank ordered by average expression. Normalized expression datasets as RSEM (RNA-seq.V2) from primary lung adenocarcinoma samples obtained through the Cancer Genome Atlas (TCGA) project (https://tcga-data.nci.nih.gov/tcga/tcgaHome2.jsp) were log_2_ (+1) transformed and the distribution of *CEBPB*, *CEBPA*, or *CDKN2A* expression was plotted as well as depicted as box plot. Correlation of *CEBPB* expression with its methylation data (HM450) and its copy number data (SNP 6.0) from TCGA data were obtained and analyzed through cBioPortal (http://www.cbioportal.org/public-portal).

### Statistical analysis

Differences between the experimental groups were tested with Student’s t-test. *P*-values of less than 0.05 were considered statistically significant.

## Results

### C/EBPβ is expressed in normal lung and adenocarcinoma cell lines

**Fig 1:**
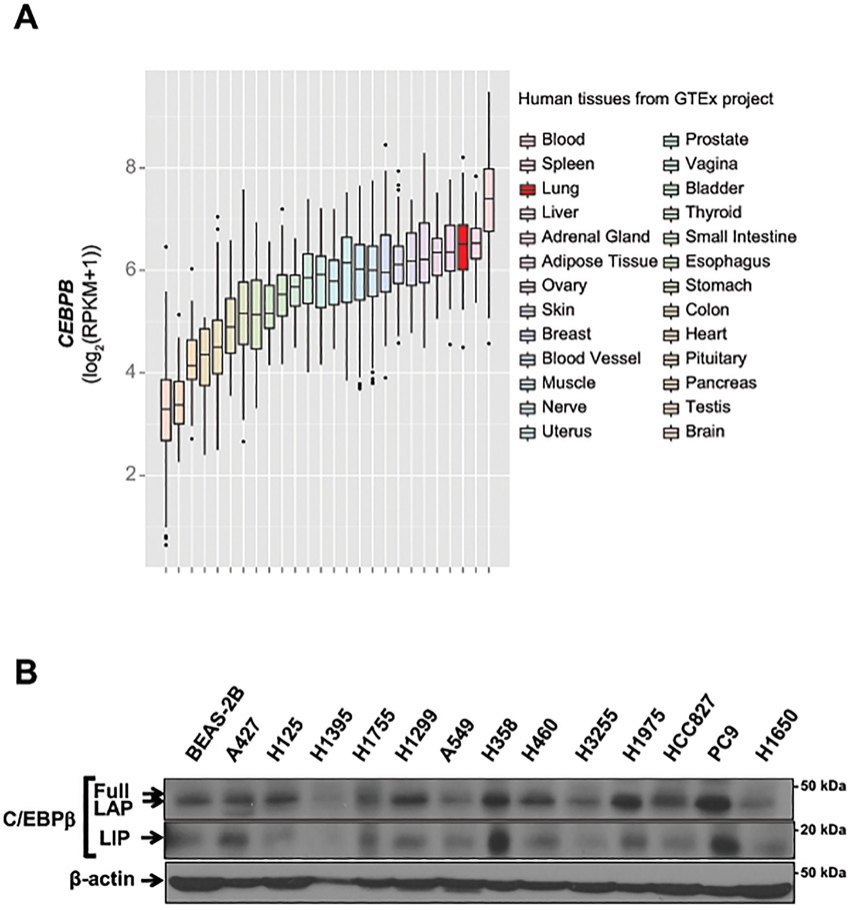
C/EBPβ is expressed in human lung cells. (A) Expression of *CEBPB* in various human tissues in the dataset from GTEx project. RPKM values were log_2_ transformed and presented as a box plot for each tissue type. (B) Protein extracts were isolated from a panel of human lung adenocarcinoma cell lines as well as an immortalized human bronchoepithelial cell line, BEAS-2B and subjected to Western blotting.

C/EBPβ regulates cellular proliferation and differentiation under normal physiological conditions. Thus, to confirm C/EBPβ is expressed in human lungs as well as rodent lungs [21, 22], we examined its expression level from various human organ systems from GTEx datasets. We found that *CEBPB* is expressed in human lung at a lower level than hematopoietic system but a level comparable to or higher than that in any other tissues examined (Fig. 1A). At a protein level, we also found that C/EBPβ expression was readily detected in BEAS-2B immortalized human bronchial epithelial cells [31] and a panel of human adenocarcinoma cell lines at variable but equivalent level (Fig. 1B). These results indicate that C/EBPβ is expressed in both benign and malignant cells of the lung.

### C/EBPβ knockout mice have normal lung appearance and structure

**Fig 2:**
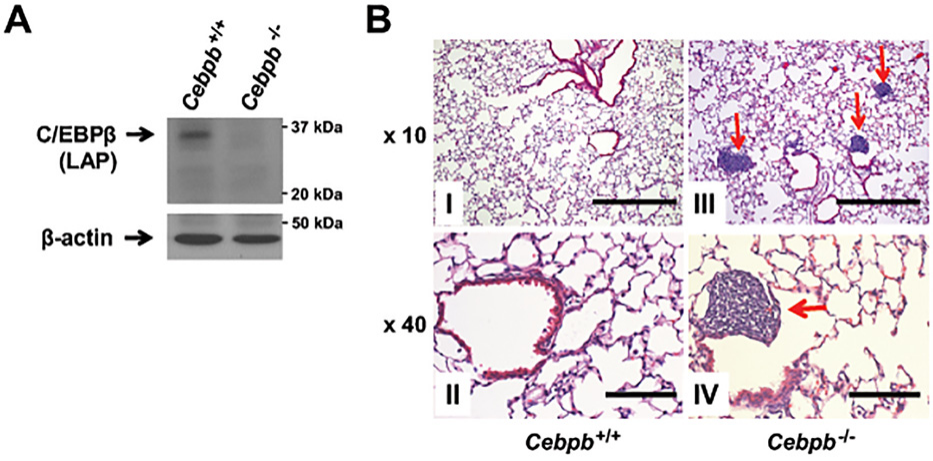
Deletion of *Cebpb* has no effect on lung structure, but leads to aggregation of lymphoid cells in the lung. (A) Immunoblots of lung extracts from *Cebpb* ^+/+^ or *Cebpb* ^*-/-*^ mice. Note that the predominant isoform expressed in mouse lungs is LAP and C/EBPβ expression in the lung is effectively lost in *Cebpb* ^*-/-*^ mice. (B) Representative images of hematoxylin and eosin staining of lungs isolated from *Cebpb* ^+/+^ (I and II) and *Cebpb* ^-/-^ (III and IV) mice. We analyzed 10 *Cebpb* ^+/+^ and 8 *Cebpb* ^-/-^ mice. Scale bars = 500 μm (I and III) and 100 μm (II and IV). Arrows indicate scattered lymphocyte aggregates in the lung parenchyma of a *Cebpb* ^*-/-*^ mouse. No gross or histologic architectural abnormalities were observed in the lungs of *Cebpb* ^*-/-*^ mice.

*Cebpb* knockout mice have been reported to manifest no histological abnormalities in the lung at perinatal stage [23, 24]. Therefore, we sought to determine whether C/EBPβ is required for development and maintenance of lung at later adult stage. Lungs were isolated from *Cebpb* knockout and control mice up to 10 weeks of age. We confirmed C/EBPβ was not expressed in the lungs of *Cebpb* knockout mice (Fig. 2A), but gross appearance of the lungs was indistinguishable from those of *Cebpb* wild type mice (data not shown). Histological analysis revealed scattered parenchymal lymphoid aggregates in 7 out of 8 (87.5%) lungs isolated from *Cebpb* knockout mice, while no such aggregates were observed in ones from wild type siblings (0/10; 0%). However, no major difference in lung parenchymal architecture was apparent between C/EBPβ knockout and wild type mice (Fig. 2B).

### C/EBPβ had no advantageous effects on cell growth in lung adenocarcinoma cells

**Fig 3:**
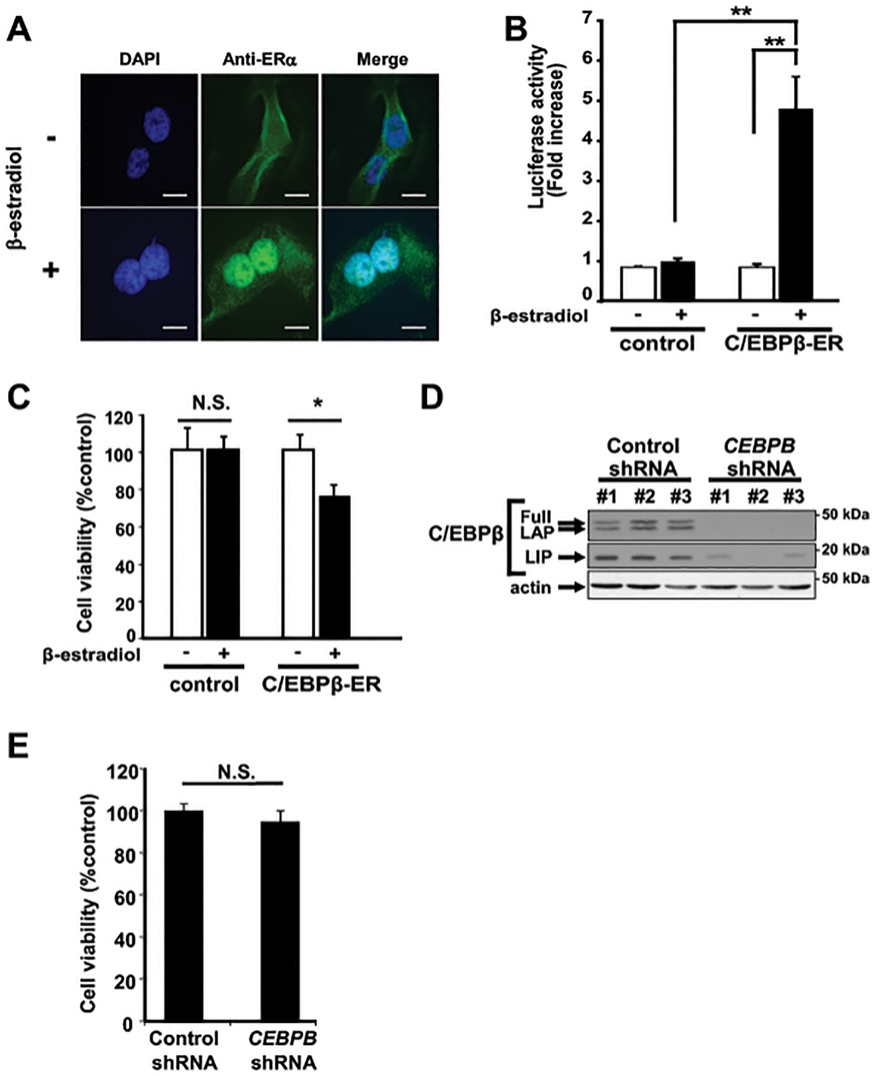
C/EBPβ fails to contribute to cell proliferation *in vitro*. (A) Fluorescent images of NCI-H358 cells stably expressing C/EBPβ-ER. Cells were treated with 1 μM β-estradiol or 0.1% ethanol (vehicle) for 24 hours and stained with anti ER antibody and visualized by Alexa Fluor 488. Scale Bar = 10 μm. (B) Luciferase assay to determine C/EBPβ transcriptional activity in NCI-H358 cells stably expressing C/EBPβ-ER and control cells. Cells were transfected with *C/ebpb0*.*3-*luc reporter and pCMV-*Renilla* control plasmid were assayed after 24 hours of treatment with 1 μM β-estradiol or vehicle control. Data represents mean ± standard deviation from three independent experiments. ** denotes *p* ≤ 0.01. (C) Cell viability determined by MTS assay. NCI-H358 cells expressing C/EBPβ-ER cells or control cells were plated in 96 well plates at 2,000 cells/well and assayed after 72 hours of incubation. Data represents mean ± standard deviation from eight independent experiments. * denotes *p* ≤ 0.05. (D) Immunoblots of C/EBPβ in NCI-H358 cells transduced with shRNA against *CEBPB*. Three clones each for control and shRNA against *CEBPB* are shown. (E) Cell viability determined by MTS assay. NCI-H358 cells transduced with shRNA against *CEBPB* or control shRNA described in (D) were plated in 96 well plates at 2,000 cells/well and assayed after 72 hours of incubation. Data represents mean ± standard deviation from three independent experiments. N.S. denotes not significant.

Expression of C/EBPβ in lung adenocarcinoma cell lines (Fig. 1B) and its reported proliferative role in epithelial cancer development [32] prompted us to hypothesize that C/EBPβ may be important for lung cancer progression. To examine proliferative effects of C/EBPβ, we generated stable NCI-H358 cell lines expressing an estrogen receptor hormone-binding domain fused to C/EBPβ (C/EBPβ-ER) or expressing the estrogen receptor hormone-binding domain alone [25, 26]. Upon stimulation with β-estradiol, C/EBPβ-ER is translocated to the nucleus (Fig. 3A) and activated(Fig. 3B)Fig. Unexpectedly, nuclear translocation of C/EBPβ-ER to the nucleus led to suppression of cell proliferation in NCI-H358 cells (Fig. 3C). Next, to examine requirement of C/EBPβ expression in lung adenocarcinoma cells, we transduced NCI-H358 cells with retrovirus containing shRNA constructs against C/EBPβ and isolated three clones. Despite greater than 90% reduction in C/EBPβ expression achieved in all clones (Fig. 3D), we observed no significant difference in cell proliferation upon C/EBPβ suppression in NCI-H358 cells (Fig. 3E). Similar results were obtained in NCI-H1975 cells (data not shown). These results suggest that C/EBPβ is not essential for and does not promote lung cancer cell growth.

### C/EBPβ activity is not mediated by EGFR signaling in lung cancer cells

**Fig 4:**
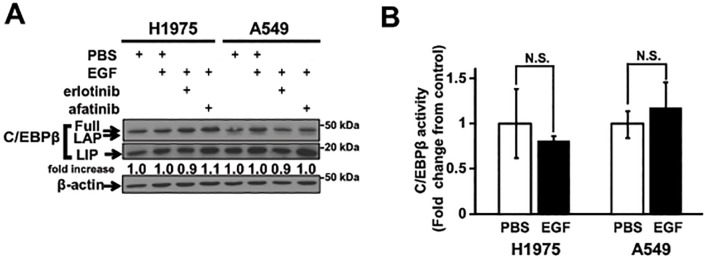
EGF does not increase LIP protein. (A) Immunoblots of C/EBPβ in NCI-H1975 (*EGFR L858R+T790M*) and A549 (*KRAS G12S*) lung adenocarcinoma cells treated with EGF in the presence or absence of EGFR kinase inhibitors for 24 hours. Changes in LIP expression are adjusted by β-actin expression and expressed as fold increase relative to that of PBS-treated cells. Note that the full and LAP forms were detected in the gel exposed for 15 seconds, whereas LIP forms were detected after 1-hourr exposure of the gel. (B) Luciferase assay to determine C/EBPβ transcriptional activity. NCI-H1975 and A549 cells transfected with *C/ebpb0*.*3-*luc reporter and pCMV-*Renilla* control plasmid were assayed after 24 hours of treatment with EGF or PBS control. Data represents mean ± standard deviation from four independent experiments. N.S. denotes not significant.

In breast cancer, EGF stimulation has been shown to reduce C/EBPβ activity by increasing LIP [33]. Therefore, we asked whether C/EBPβ activity can be regulated by the EGFR signaling pathway in the lung cancer cells. To this end, we examined two cell lines, NCI-H1975 and A549 cells, which harbor the *EGFR L858R+T790M* double mutations and *KRAS G12S* mutation, respectively. When these cells were treated with EGF in the presence or absence of EGFR tyrosine kinase inhibitors (erlotinib or afatinib), no apparent changes in expression of C/EBPβ LIP isoform were detected (Fig. 4A). Consistent with these results, EGF stimulation led to no significant change in C/EBPβ activity determined by luciferase assay in these two cell lines (Fig. 4B). Therefore, it appears unlikely that EGFR signaling mediates C/EBPβ activity in lung cancer cells.

### C/EBPβ is dispensable for lung tumorigenesis in the EGFR mutant-driven murine lung cancer model

**Fig 5:**
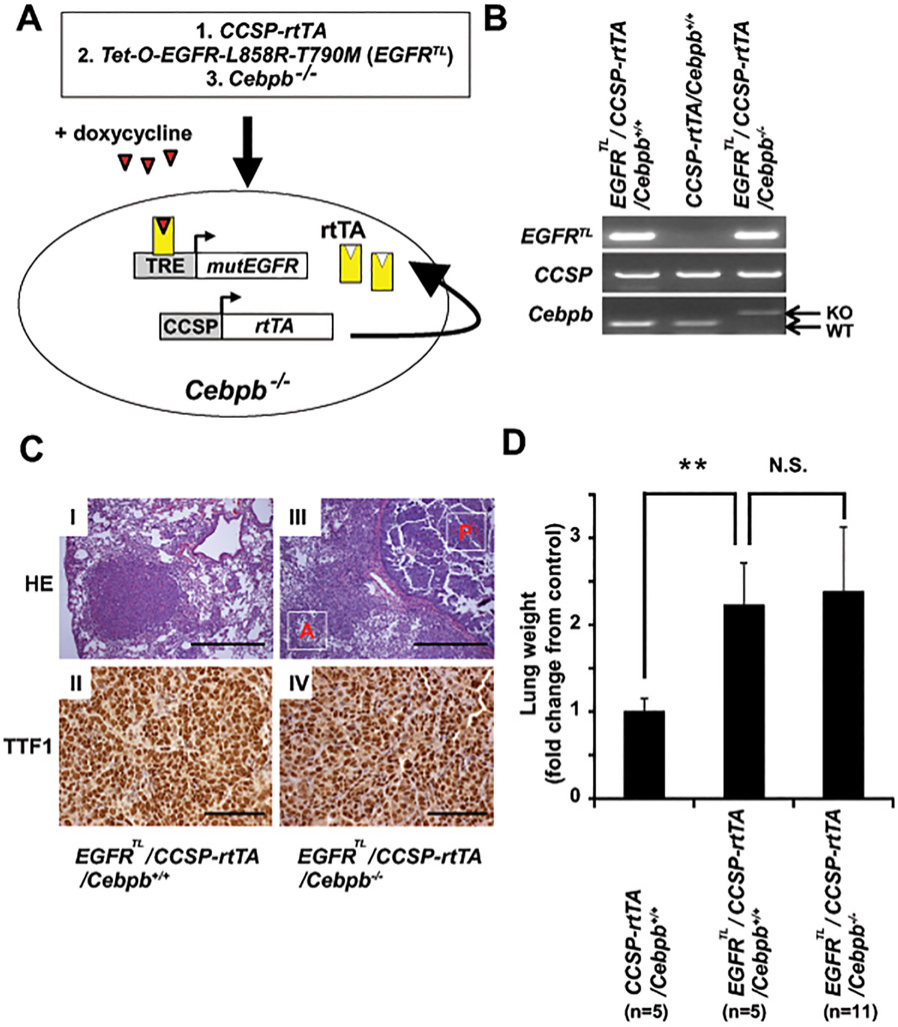
Deletion of *Cebpb* does not affect tumor burden *in vivo*. (A) Scheme of the strategy generating lung specific inducible *EGFR-TL* transgenic mice with *Cebpb* knockout background. (B) Genotype of *EGFR* ^*TL*^ */CCSP-rtTA/Cebpb* ^+/+^, *CCSP-rtTA/Cebpb* ^+/+^ and *EGFR* ^*TL*^ */CCSP-rtTA/Cebpb* ^-/-^ mice. (C) Representative images of H&E staining and immunohistochemistry of lungs isolated from *EGFR* ^*TL*^ */CCSPrtTA/Cebpb* ^*+/+*^(I and II) or *EGFR* ^*TL*^ */CCSPrtTA/Cebpb* ^*-/-*^(III and IV). Lung sections were stained with H&E (I and III) and anti-TTF1 (II and IV) antibodies. “A” indicates an area with bronchioalveolar growth pattern and “P” indicates an area with bronchial papillary growth pattern. (D) Lung weight isolated from mice treated with doxycycline for 8–12 weeks. We analyzed 5 *CCSP-rtTA/Cebpb* ^*+/+*^, 5 *EGFR* ^*TL*^ */CCSP-rtTA/Cebpb* ^*+/+*^, and 11 *EGFR* ^*TL*^ */CCSP-rtTA/Cebpb* ^*-/-*^ mice. Data represent mean ± standard deviation. ** denotes *p* ≤ 0.01. N.S. denotes not significant.

Our results from lung cancer cell lines suggest that C/EBPβ in tumor cells may not drive cell growth. However, these results should be confirmed *in vivo*. In addition, it is possible that C/EBPβ play an important role to maintain tumor microenvironment. Therefore, we generated the inducible *EGFR T790M-L858R* transgenic mouse model in *Cebpb* knockout background. We crossed two established mouse models, the lung specific EGFR-L858R-T790M (*EGFR* ^*TL*^)/*CCSP-rtTA* bi-transgenic mice [27], and the C/EBPβ conventional knockout mice (*Cebpb* ^*-/-*^) (Fig. 5A and 5B) [28]. Consistent with our previous report [27], TTF-1 positive lung adenocarcinomas developed in *EGFR* ^*TL*^ */CCSP-rtTA*/*Cebpb* ^*+/+*^mice (Fig. 5C; I and II) as well as in *EGFR* ^*TL*^ */CCSP-rtTA*/*Cebpb* ^*-/-*^mice (Fig. 5C; III and IV) when treated with doxycycline for 10 weeks. By histological analysis, the lung adenocarcinomas in *EGFR* ^*TL*^ */CCSP-rtTA*/*Cebpb* ^*-/-*^ mice tended to demonstrate a more central bronchial papillary growth pattern (Fig. 5C; III), whereas the adenocarcinomas in *EGFR* ^*TL*^ */CCSP-rtTA*/*Cebpb* ^*+/+*^mice tended to have a more peripheral distribution with solid and bronchioalveolar growth features (Fig. 5C; I). However, no significant differences were noted regarding overall tumor burden as reflected by gross lung weights (Fig. 5D). These data suggest that C/EBPβ is dispensable for lung tumorigenesis in EGFR-driven murine lung cancer.

### C/EBPβ is neither overexpressed nor downregulated in human lung adenocarcinoma

**Fig 6:**
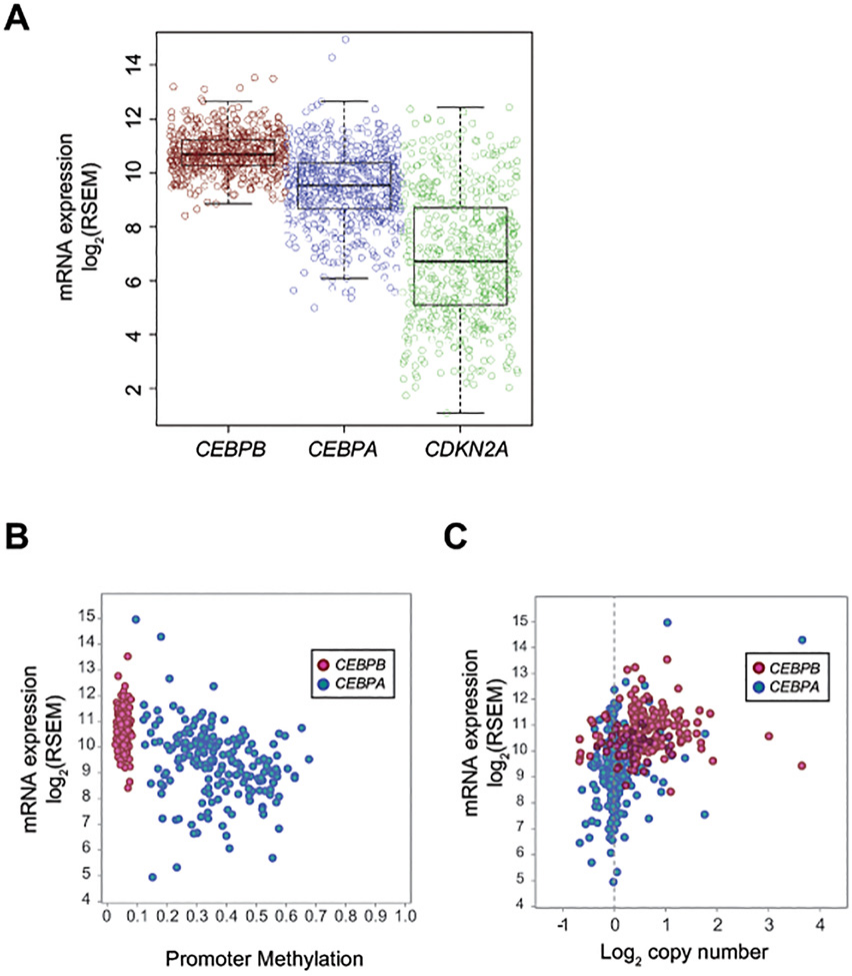
C/EBPβ is not altered in human lung adenocarcinoma. (A) mRNA expression of *CEBPA*, *CEBPB*, and *CDKN2A* in TCGA lung adenocarcinoma dataset. RSEM values obtained from TCGA data were log_2_ transformed and depicted as individual dots representing a sample and box plot. (B) Methylation status in *CEBPA* and *CEBPB* promoter regions in TCGA lung adenocarcinoma dataset. Scatter plot depicts mRNA expression in Y-axis and methylation level of the promoter region in X-axis of *CEBPA* and *CEBPB* genes. (C) Copy number estimates of *CEBPA* and *CEBPB* gene loci in TCGA lung adenocarcinoma dataset. Scatter plot depicts mRNA expression in Y-axis and copy number estimates in log_2_ scale in X-axis of *CEBPA* and *CEBPB* genes.

Lastly, we sought to determine whether C/EBPβ expression is altered in human lung adenocarcinoma using data from The Cancer Genome Atlas (TCGA). We confirmed that two tumor suppressors, *CEBPA* and *CDKN2A*, were downregulated in a subset of human lung adenocarcinomas as previously described [34, 35]. Compared to these two genes, expression of *CEBPB* was maintained in these tumor samples (Fig. 6A). Next, we examined the methylation status of the *CEBPA* and *CEBPB* promoters. The *CEBPB* promoters were unmethylated in all lung adenocarcinoma samples with available methylation data (n = 185), whereas the *CEBPA* promoter regions were frequently aberrantly methylated, consistent with previous results [36] (Fig. 6B). By examining the matched copy number data, we found that the CEBPA and CEBPB loci were not subject to frequent deletion (Fig. 6C). Taken together, these data suggest that expression of C/EBPβ is not altered in human lung adenocarcinoma.

## Discussion

C/EBP proteins consist of a group of basic-leucine zipper (bZIP) transcription factors which are involved in the regulation of important functions such as proliferation and differentiation, survival and apoptosis, oncogene-induced senescence and tumorigenesis, inflammation, immunity, and metabolism [37]. In this study, we investigated the role of C/EBPβ on lung adenocarcinomagenesis.

C/EBPα and C/EBPβ reportedly play partly overlapping roles in some tissues. Knock-in mice where the C/EBPα gene function is replaced by that of C/EBPβ partly rescues the hematopoietic phenotype of the C/EBPα knockout mice, providing evidence that C/EBPβ could compensate for loss of C/EBPα in hematopoiesis [38]. However, C/EBPβ seems to have more complex roles as it can act as either a promoter or an inhibitor to cell proliferation under the different cellular contexts or tissues [33]. C/EBPβ is up-regulated during macrophage differentiation [18], indicating that C/EBPβ shows anti-proliferative and differentiation-inducing function similar to C/EBPα. On the other hand, partial hepatectomy leads to increased expression of C/EBPβ, suggesting that C/EBPβ is required for hepatocyte proliferation [15]. Although both C/EBPα and C/EBPβ are expressed in pulmonary cells in rodents [21, 22] and humans (Fig. 1A), it is unlikely that C/EBPβ is involved in normal lung homeostasis as there was no striking difference in lung architecture between wild type and *Cebpb* knockout mouse at adult stage (Fig. 2B). However, C/EBPβ can be upregulated in the lung together with C/EBPδ when challenged with acute-phase stimuli [39]. In this regard, it is notable that scattered parenchymal lymphoid aggregates were frequently observed in lungs isolated from *Cebpb* knockout mice (Fig. 2B: IV) possibly due to upregulation of IL-6 in *Cebpb* knockout mice [28]. As infiltrated lymphocytes may regulate neutrophil recruitment during acute lung injury [40], it is possible that C/EBPβ may play a role to resolve acute inflammation by suppressing accumulation of lymphoid cells. These questions remain to be addressed.

Based on our observation that C/EBPβ was expressed in lung adenocarcinoma cell lines (Fig. 1B), we hypothesized that C/EBPβ may contribute to lung cancer development. Although C/EBPα has been established as a cell cycle inhibitor/tumor suppressor [41], several lines of evidence suggest that the role of C/EBPβ in tumorigenesis seems to be tissue-specific. *All-trans* retinoic acid induces differentiation of acute promyelocytic leukemia (AML) cells via C/EBPβ expression [25]. Overexpression of C/EBPβ in HepG2 hepatocellular carcinoma cells strongly inhibits tumor cell proliferation. On the other hand, C/EBPβ is highly expressed and was associated with tumor progression in colorectal and ovarian cancers [42, 43]. Furthermore, deletion of *Cebpb* in keratinocytes protects carcinogen-induced skin tumorigenesis [32]. In the current study, neither overexpression of nuclear C/EBPβ nor suppression of *CEBPB* expression showed evidence that supports tumor promoting or suppressive role of C/EBPβ (Fig. 3). Furthermore, unlike in breast cancer cells [33], LIP protein and C/EBPβ activity upon EGF stimulation were unchanged in lung adenocarcinoma cell lines (Fig. 4), indicating that C/EBPβ may not be involved in proliferation in response to growth factor stimulation. These *in vitro* data was supported by our *in vivo* mouse model (Fig. 5) and clinical data (Fig. 6).

Notably, however, lung adenocarcinomas from *EGFR* ^*TL*^ */CCSP-rtTA*/*Cebpb* ^*-/-*^ mice show a propensity to form papillary tumors in the bronchial airways (Fig. 5C; III). Given that there are no gross or histologic differences in lung architecture or epithelial differentiation observed in *Cebpb* knockout lungs [24], it is plausible that environmental alternations led by disruption of *Cebpb* may have affected this histological phenotype. One possibility is that IL-6 may be involved in cell fate conversion efficiency. As described above, IL-6 is shown to be upregulated in *Cebpb* knockout mice [28] and can lead to regeneration of airway ciliated cells from basal stem cells [44]. In addition, inflammatory cytokines including IL-6 can promote de-differentiation of tumor cells into progenitor cells in hepatocellular carcinoma [45]. Therefore, alterations of cytokine expression induced by deletion of *Cebpb* may have influenced the observed cell types in lung adenocarcinomas.

In summary, we confirmed that overall lung architecture was maintained in *Cebpb* knockout mice. While C/EBPβ was expressed in human adenocarcinoma cell lines, neither overexpression of nuclear C/EBPβ nor knockdown of *CEBPB* had significant effect on cell proliferation. Deletion of *Cebpb* had no impact on lung tumor burden in a murine lung cancer model. Expression of *CEBPB* was not altered in human lung cancer samples. Taken together, our data indicate that C/EBPβ largely is not essential for lung homeostasis or development of lung adenocarcinoma.
